# Emerging Role of Mucolipins TRPML Channels in Cancer

**DOI:** 10.3389/fonc.2020.00659

**Published:** 2020-04-28

**Authors:** Giorgio Santoni, Matteo Santoni, Federica Maggi, Oliviero Marinelli, Maria Beatrice Morelli

**Affiliations:** ^1^Section of Immunopathology, School of Pharmacy, University of Camerino, Camerino, Italy; ^2^Medical Oncology Unit, Hospital of Macerata, Macerata, Italy; ^3^Department of Molecular Medicine, University of Rome Sapienza, Rome, Italy; ^4^School of Biosciences and Veterinary Medicine, University of Camerino, Camerino, Italy

**Keywords:** cancer, tumor progression, ion channels, transient receptor potential channels, mucolipins

## Introduction

At the end of 2018, Faris and colleagues published an article in Cancers entitled “Endolysosomal Ca^2+^ signaling and cancer hallmark: two pore channels on the move and TRPML1 lags behind!” emphasizing the emerging role of two pore channels (TPC) in cancer, in contrast to that of comparable transient receptor potential mucolipin channel 1 (TRPML1) the role of which in cancer was still obscure (1).

TPCs and TRPMLs are endolysosomal channels regulating the autophagy/lysosome system, which is strongly associated both with cancer progression and cancer escape from immunosurveillance. A role for TPCs in cancer cell invasiveness and metastasis has been established (2–4). Instead, regarding the TRPML channels, the evidence for their role in cancer was scarce until recently. At present this gap has been filled and findings until now are summarized in Table 1.

**Table 1:** TRPMLs and cancer.

	**Tumor type**	**Effect**	**Reference**
**TRPML1**			
High expression	Head & Neck Bladder	HRAS activity ↑ Patient prognosis ↓	(5)
High expression	Melanoma	MAPK and mTORC1 ↓ Survival and proliferation ↑	(6)
High expression	High grade NSCLC	Migration, proliferation and autophagy ↑	(7)
High expression	PDAC	OS and PFS ↓	(8)
High expression	TNBC	Cancer growth ↑	(9)
High expression	Endometrial	Lysosomal pH ↑ Imidazole cytotoxicity ↓	(10)
Loss expression or reduction	GBM	OS ↓	(11)
Activation	GBM	Cell viability ↓ Apoptosis ↑	(11)
**TRPML2**			
High expression	GBM	Cell viability ↑ Apoptosis ↓	(12)
Concurrent gene in 16-gene signature	Breast	Survival ↑	(13)
Downregulation due to DNA methylation	Acute Lymphoblastic Leukemia	Pro-tumoral effects ↑	(14)
**TRPML3**			
Decreased gene expression	High-risk PDAC patients, KIRK	Protective role, OS ↑	(15)
Decreased gene expression	Adenocortical, Breast invasive, Endometrial, Kidney Renal Papillary Cell, Colon, Lung, Lung Squamous Cell, Rectal, Stomach, Pheochromocytoma, Paraganglioma, Thymoma, Uterine Carcinosarcoma	Not investigated	(15)

*(↑), increase; (↓) impairment*.

## TRPML Channels

TRPML1, TRPML2, and TRPML3 are non-selective cation channels belonging to the TRP channel family. These channels are encoded by *MCOLN* genes (*MCOLN1, MCOLN2*, and *MCOLN3*) located, respectively, on human chromosome 19 (19p13.2-13.3), chromosome 1 (1p22.3) and chromosome 1 (1p22.3).

The founding member TRPML1 is expressed in a number of tissues including adrenal gland, lung, bladder and placenta as well as in thymus, spleen and immune cells (16, 17). Mutations in this channel have been identified as responsible for the phenotype of Mucolipidosis type IV, a severe lysosomal storage disorder that displays cognitive, linguistic, visual and motor deficits (18–20). TRPML2 mRNA is mainly detected in lymphocytes and other cells of the immune system (21–24). No human or mouse disease phenotype has been demonstrated for this channel. TRPML3 is expressed in cochlear and vestibular sensory hair cells and melanocytes (25). Two spontaneous gain-of-function mutations in TRPML3 cause hearing loss and coat color dilution in mice (26–30). These mutations are known as varitint-waddler mutations (A419P and I362T).

TRPML1 is mainly localized in the lysosomes, where it promotes the cation efflux into the cytosol (31). In this way TRPML1 is involved in lysosomal storage, transportation and pH homeostasis. Indeed, TRPML1 mutations affect lysosomal storage and lysosomal impairment is responsible for autophagy defects. TRPML1 can also be negatively regulated by the target of rapamycin (TOR) with a consequent autophagy decrease supporting a central role of TRPML1 in this process (32). Moreover, oxidative stress through TRPML1 activation triggers autophagy to re-establish cellular redox homeostasis (33). TRPML1 regulates exocytosis of intracellular contents via the endosomal-lysosomal pathway (34, 35). In addition, TRPML1 plays a role in metal ion homeostasis (36–39). TRPML2 and TRPML3 are expressed in early endosomes, late endosomes and lysosomes (40, 41); TRPML2 is also active in recycling endosomes (42, 43). It plays a direct role in chemokine secretion and thus modulates the inflammatory response. TRPML3 could be required for the autophagic process. Indeed, it is suggested that this channel, changing subcellular localization, provides Ca^2+^ during the autophagosome formation but the underlying mechanisms are not completely known (40, 44, 45).

TRPMLs are activated by one of the major components of endolysosomal membranes, the phosphatidylinositol-3,5-biphosphate (PtdIns(3,5)P2) (25, 46–49). Moreover, TRPML1 has an intraluminal loop whose protonation stimulates channel activation (28, 37, 50). It is inhibited by phosphatidylinositol-4,5-biphosphate (PtdIns(4,5)P2), sphingomyelins, and lysosomal adenosine (48, 49). Na^+^ removal or less acidic/neutral pH activates TRPML3 and TRPML2, respectively (42, 43). Among the synthetic activators that are currently available, ML-SA1 activates TRPML1, TRPML2, and TRPML3 in humans; ML2-SA1 is TRPML2 specific; MK6-83 activates TRPML1 and TRPML3 (25, 43, 46, 48). There are several synthetic inhibitors (ML-SIs); however, they are unable to discriminate the different TRPML isoforms (33, 35).

## TRPML1 and Cancer

In the last year several reports have linked increased TRPML1 expression to cancer. Among all, Jung et al. (5) demonstrated a correlation between human cancers with activating *HRAS* mutations and increased TRPML1 expression. In head and neck squamous cell carcinoma and bladder urothelial carcinoma, that display high rates of *HRAS* mutations, TRPML1 expression inversely correlates with patient prognosis. It was demonstrated that the enhancement in TRPML1 expression and activity in HRAS-driven cancer cells is required for localization of cholesterol in the plasma membrane. Indeed, its mislocalization from the plasma membrane to endolysosomes leads to loss of oncogenic HRAS from the cell surface and inhibition of downstream signaling. At the same time, TRPML1 inhibition decreases extracellular signal-regulated kinase (ERK) phosphorylation levels and cell proliferation.

The analysis of specific tumor types indicates that TRPML1 may exert different, and even opposite, functional activity in controlling tumor growth and progression.

Kasitinon et al. (6) have demonstrated that melanoma cells show a higher TRPML1 expression than normal melanocytes. *In vitro* and *in vivo* experiments revealed that loss of TRPML1 reduces melanoma cell growth in a xenograft model, but does not affect human melanocyte cell growth. However, in contrast to Jung et al. (5), Kasitinon et al. (6) reported that loss of TRPML1 leads to high phosphorylated MET and ERBB3 levels and thus MAPK activation in melanoma cells. The deregulation of these signaling pathways could be attributed to the reduced lysosomal degradation of growth factor receptors and the unique nature of melanomas. Moreover, TRPML1-deficient melanoma cells show, as a consequence of activated mTORC1 signaling, increased protein synthesis, unfolded protein response activation and cell death.

Pancreatic ductal adenocarcinoma (PDAC) patients characterized by worse overall survival and progression-free survival express high TRPML1 levels (8). In support of TRPML1 pro-tumoral role, its knockdown blocks the proliferation of PDAC cells *in vitro* and reduces the formation and growth of tumors in *in vivo* mouse models (8).

TRPML1 is upregulated in triple-negative breast cancer (TNBC) and its genetic down-regulation or pharmacological inhibition, suppresses TNBC growth. TRPML1 channel is involved also in controlling TNBC development by regulating mTORC1 activity and lysosomal ATP release via purinergic signaling pathways (9).

In human endometrial adenocarcinoma (HEC-1B) cells, TRPML1 overexpression inhibits vacuole formation and cell death induced by the lysosomotropic agent imidazole. In contrast, TRPML1 knockdown increases cell death induced by the same treatment (10). Thus, it was supposed that TRPML1 is able to protect against this kind of drug by regulating the pH of acidic organelles.

In contrast to the above-mentioned tumors, TRPML1 expression is decreased in human non-small-cell lung carcinoma (NSCLC) tissues compared to normal lung tissues (7). However, channel expression increases in advanced stages. These data suggest that *MCOLN1* gene deletion supports tumorigenesis as a consequence of increased oxidative stress and genetic instability, while TRPML1 may confer a survival advantage in advanced tumors (7). In accordance with these findings, *in vitro* studies have demonstrated that TRPML1 silencing suppresses migration and proliferation of A549 and H1299 cancer cell lines. Instead, in transfected NSCLC cell lines, TRPML1 overexpression promotes autophagy. Collectively these findings indicate that, the loss of TRPML1 leads to a reduction in autophagic activity, which is essential for NSCLC survival and proliferation, while increase in TRPML1 expression is needed to keep metabolism and energy requirements of the tumor in balance (7).

In glioblastoma (GBM) patients loss or reduction of TRPML1 correlated with short survival, thus acting as negative prognostic factor (11). *In vitro* studies supported that TRPML1 activation, due to the agonist MK6-83, reduces cell viability and induces apoptosis (11). Strong oxidative stress may damage cellular constituents (e.g., mitochondria) permanently hence acting as a death signal. However, the generation of low ROS levels can act as survival signal, by triggering autophagy (33). In this regard, since the primary source of endogenous ROS are mitochondria, treating glioma cells with carbonyl cyanide m-chlorophenylhydrazone (CCCP), that is able to induce oxidative stress (33), stimulates a TRPML1-dependent autophagy, as demonstrated by the ability of the autophagic inhibitor bafilomycin-A1, the TRPML1 inhibitor sphingomyelin, and *MCOLN1* silencing, to inhibit the CCCP-mediated effects (11).

## TRPML2 and Cancer

A link between TRPML2 expression and cancer has been investigated in different tumor types. A 16-gene signature, including *MCOLN2* was found to be associated with distinct survival patterns in breast cancer patients (13).

In acute lymphoblastic leukemia patients, aberrant methylated regions are associated with altered expression of a set of genes (14). Among them, *MCOLN2*, affecting cell cycle processes, lymphocyte activation and apoptosis, has been found hypermethylated and downregulated. The function of TRPML2 in B lymphocytes is unclear; however, its transcriptional regulation by the B-cell lineage activator PAX5 and its expression in early immature B cells, may support for a role played by TRPML2 in the development of acute lymphoblastic leukemia.

Furthermore, the analysis of the transcriptome in colorectal cancer (CRC) showed that TRPML1 and TRPML2, detected in both normal and cancer cells, are significantly decreased in CRC cells. Noteworthy TRPML2 is dramatically down-regulated (51).

In GBM, TRPML2 was found to play different role than TRPML1 (12). TRPML2 is expressed in astrocytes, glioma tissues and high-grade glioma cell lines, with increased expression in GBM compared to pylocitic astrocytoma (grade I) (12). TRPML2 knockdown reduces proliferation by abrogating protein kinase B (AKT)/ERK1/2 phosphorylation and induces apoptosis in glioma cell lines. Overall, a pro-tumorigenic role of TRPML2 has been suggested (12).

## TRPML3 and Cancer

The role of MCOLN3 in cancer has been poorly investigated. By integrative analysis of multiple datasets, the *MCOLN3* gene has been included in a 9-gene signature that is able to predict the overall survival of pancreatic cancer patients. The high-risk group expressing *MCOLN3* is enriched with multiple oncological signatures and pathways involved in cancer aggressiveness and associated with significantly lower levels of CD4^+^ T cell infiltration (15).

Moreover, the data analysis, using The Cancer Genome Atlas (TCGA) database, showed that *MCOLN3* is downregulated in several cancer types, and it is associated with a relatively better survival in kidney renal clear cell (15).

## Discussion

Heterogeneity of TRP channels expression is a feature of cancer cells and changes in their expression and functions are associated with neoplastic transformation and progression. We have just started to understand the genetic and epigenetic mechanisms controlling TRPML channel expression and endolysosomal Ca^2+^ signaling which seem to regulate cancer cell behavior. Nevertheless, several issues remain unsolved. How are TRPML channels regulated in cancer cells? TRPML homo/heteromerization or genic compensation is physiologically relevant in normal cells (52, 53), but what about in cancer cells? Currently it remains unclear in what aspects such interactions are physiologically relevant and what the physiological consequences would be when interfering with these interactions (17, 54). Moreover, which are the main TRPML ligands in cancer cells? Which is the contribution of immune cells expressing the TRPML channels in tumor microenvironment? And do the TRPML channels play a role in tumor escape?

Several studies describe that TRPML1 and TRPML2 represent suitable molecular targets in cancer. Thus, genetic manipulation or pharmacological modulation of TRPML expression and function could be regarded as a promising alternative anti-cancer therapy.

A genetic approach targeting TRPML expression could represent a good strategy. However, there is a potential for off-target effects linked with the ubiquitous expression of TRPML1 and the involvement of TRPML2 in immune functions. Moreover, loss of function mutations in *MCOLN1* are responsible for the onset of neurological disorders. As suggested by Jung and Venkatachalam, the use of antisense oligonucleotides, unable to cross the blood-brain-barrier, may circumvent this problem (55).

The therapeutic approach is also hampered by a relative lack of TRPML selective agonists or antagonists; indeed, TRPML channels share high sequence similarity of about 75% at amino acid levels (56). Given the ability of PI(3,5)P2 to activate TRPMLs, researchers have also focused their efforts on the regulation of the PIKfyve/PI(3,5)P2 pathway in cancer. Studies conducted on hepatic cancer cell lines demonstrated the effectiveness of the inhibitor of PI(3,5)P2 biosynthesis, YM201636, on attenuating cancer cell proliferation (57). Treatment with the PIKfyve inhibitor apilimod is able to induce cytotoxic effects in B-cell non-Hodgkin lymphoma and TFEB, the master transcriptional regulator of lysosomal biogenesis involved in a feedback loop with TRPML1 (58), regulates apilimod sensitivity (59).

Another level of complexity is related to the molecular mechanisms and signaling pathways acting both up- and down-stream of TRPMLs. A role of mTORC1 and RAS-ERK as well as Akt/PKB/ERK in TRPML1 (6, 60) and TRPML2-expressing tumor cells (12) has been proposed, although there are divergent findings in different cancer cell types. These opposite results may reflect distinct effects of TRPML on different cancer cells that could be due to distinctive mutations, for example HRAS signaling is mutated in head and neck cancer cells but not in melanoma cells (6, 61).

Overall, mucolipin targeting might represent a novel and innovative approach in the prognosis and therapy of different cancer types. Obviously, more in-depth studies are needed to completely reveal their potential in cancer therapy.

## Author Contributions

GS wrote the manuscript. MM, MS, OM, and FM revised the manuscript. MM and OM re-examined and revised the manuscript.

## Conflict of Interest

The authors declare that the research was conducted in the absence of any commercial or financial relationships that could be construed as a potential conflict of interest.
